# Association Between Oxytocin Receptor Genotype, Maternal Care, and Eating Disorder Behaviours in a Community Sample of Women

**DOI:** 10.1002/erv.2486

**Published:** 2016-11-14

**Authors:** Nadia Micali, Marta Crous‐Bou, Janet Treasure, Elizabeth A. Lawson

**Affiliations:** ^1^ Eating and Weight Disorders Program, Department of PsychiatryIcahn School of Medicine at Mount SinaiNew YorkNYUSA; ^2^ Mindich Child Health and Development InstituteIcahn School of Medicine at Mount SinaiNew YorkNYUSA; ^3^ Institute of Child HealthUniversity College LondonLondonUK; ^4^ Channing Division of Network Medicine, Department of MedicineBrigham and Women's Hospital and Harvard Medical SchoolBostonMAUSA; ^5^ Department of EpidemiologyHarvard T. H. Chan School of Public HealthBostonMAUSA; ^6^ Eating Disorders Research Unit, Psychological MedicineInstitute of Psychiatry, King's College LondonLondonUK; ^7^ Neuroendocrine UnitMassachusetts General Hospital and Harvard Medical SchoolBostonMAUSA

**Keywords:** ALSPAC, eating disorders, oxytocin, gene–environment interaction

## Abstract

This study aimed to investigate associations between oxytocin receptor gene (*OXT‐R*) polymorphisms (rs53576 and rs2254298), their interaction with maternal care (GxE), and ED behaviours in a community sample. We studied 3698 women from the Avon Longitudinal Study of Parents and Children (ALSPAC) who participated in a two‐phase prevalence study of lifetime ED and had genotype data. The GG rs53576 genotype was associated with binge eating and purging, and the rs2254298 AG/AA genotype with restrictive eating lifetime. In addition, the rs2254298 AG/AA genotype interacted with poor maternal care to increase the odds of binge eating and purging (odds ratio = 4.40 (95% confidence intervals: 1.11–17.4)). This study replicates previous findings of an association between *OXT‐R* polymorphisms and ED, and it is the first to show an interaction between *OXT‐R* genotype and poor maternal care. As such, these findings highlight the important role of oxytocin in understanding the pathophysiology of ED. © 2016 The Authors European Eating Disorders Review published by Eating Disorders Association and John Wiley & Sons Ltd

## Introduction

There is increasing evidence that variations in levels and response to the neuropeptide oxytocin are relevant to several psychiatric disorders. In particular, research on the role of oxytocin on cognition and behaviour has focused on autism spectrum disorders, social behaviour, and emotional responses both in animals and humans (Brune, 2012; Kawamura et al., 2010; Kirsch, 2015; LoParo & Waldman, 2015; Saphire‐Bernstein, Way, Kim, Sherman, & Taylor, 2011).

In addition to differences in oxytocin levels, recent research has focused on sensitivity to oxytocin. Variations in sensitivity to oxytocin are thought to be the result of genetic and epigenetic differences in the oxytocin receptor (*OXT‐R*) gene. Genetic polymorphisms of the *OXT‐R* have received much attention in relation to prosocial behaviour, empathy, and neuropsychiatric disorders including autism spectrum disorders; in particular, the most studied single nucleotide polymorphisms (SNPs) of the *OXT‐R* gene are rs53576 and rs2254298. For example, the rs53576 GG genotype has been associated with higher levels of empathy, lower levels of depression, and pro‐social behaviour (J. Li et al., 2015; Rodrigues, Saslow, Garcia, John, & Keltner, 2009). On the other hand, some studies have failed to show similar associations (J. Li et al., 2015; Lucht et al., 2009; Wu, Li, & Su, 2012). Similarly, rs2254298 genotypes have been associated with sociability, affect, and temperament (Brune, 2012) and the GG genotype in particular with lower plasma levels of circulating oxytocin (Feldman et al., 2012). Although contrasting findings have been reported as to which rs2254298 genotype might be associated with psychopathology, there is evidence that having at least one A allele (vs. G) on a 7‐SNP haplotype block containing rs2254298 is associated with higher depression scores (Kawamura et al., 2010).

Given the role of oxytocin in affiliation, a number of studies have addressed gene–environment interactions in relation to *OXT‐R*. Interactions between *OXT‐R* polymorphisms and childhood maltreatment have been shown in two studies, with evidence of higher levels of emotional dysregulation in one study (Bradley et al., 2011) and depression in a second study (McQuaid, McInnis, Stead, Matheson, & Anisman, 2013) in carriers of the rs53576 GG genotype (Bradley et al., 2011) or G allele (McQuaid et al., 2013) who also experienced childhood maltreatment.

Increasing evidence has highlighted a key role for oxytocin in feeding behaviour (Leng et al., 2008) and appetitive control (Sabatier, Leng, & Menzies, 2013). Early experiments showing increases in food intake and weight as a result of brain lesions affecting hypothalamic nuclei containing oxytocin (Leibowitz, Hammer, & Chang, 1981) were compounded by studies in animals and humans demonstrating that exogenous oxytocin administration reduces caloric consumption, particularly of palatable foods (Arletti, Benelli, & Bertolini, 1990; Lawson et al., 2015; Maejima et al., 2015; Olson et al., 1991).

Given the role of oxytocin in feeding and eating behaviour, oxytocin pathways have recently become a focus of study in eating disorders (ED) (Monteleone, Scognamiglio, Volpe, Di Maso, & Monteleone, 2016; Tortorella et al., 2014). Low basal unstimulated serum oxytocin levels have been demonstrated in low‐weight, partially recovered, and weight‐recovered women with anorexia nervosa (AN) compared to healthy controls, suggesting a potential etiologic role (Afinogenova Y, in press; Lawson et al., 2011; Lawson et al., 2012). Furthermore, postprandial levels of oxytocin were correlated with the severity of ED psychopathology and fMRI hypoactivation of relevant homestatic and hedonic food motivation neural circuitry in AN (7). These data indicate that abnormalities in oxytocin pathways persist in AN despite weight recovery and are associated with abnormal neural activation, thoughts, and behaviours related to eating.

Two recent studies have investigated associations between *OXT‐R* genetic variants and ED. In particular, a Korean study showed a positive association between the G allele of rs53576 and bulimia nervosa (BN) in a clinical sample of 90 women with BN compared to controls (*n* = 103) (Kim, Kim, Kim, Shin, & Treasure, 2015). Amongst patients with AN, AN (weight‐recovered), BN and controls (overall *n* = 124), subjects with rs53576 and rs2254298 GG/GG haplotype had lower scores on overall ED psychopathology measures, less body shape and food preoccupation, fewer obsessive compulsive behaviours, and lower anxiety (Acevedo, Valencia, Lutter, & McAdams, 2015). A recent investigation of women from the Avon Longitudinal Study of Parents and Children (ALSPAC) sample showed that those with the rs53576 GG genotype differed in terms of dietary intake and were more likely to have endorsed having ever self‐induced vomiting or used laxatives for weight loss on a questionnaire at enrollment in pregnancy (Connelly et al., 2014). Given the lack of significant ‘hits’ in large genome wide studies (GWAS) of AN, a targeted approach to specific polymorphisms might still prove fruitful in our quest to clarify the neurobiology of ED.

Given a recent data collection of lifetime interview data on ED amongst ALSPAC women (about 20 years after enrollment), hence more precise phenotyping, and lack of availability of information on other ED behaviours (binge‐eating, restrictive eating) in the previous study, we aimed to extend previous findings by focusing on lifetime interview‐collected ED behaviours. This approach allowed us to investigate narrow ED behavioural phenotypes that might be more closely associated with specific genotypes, compared to heterogeneous diagnostic syndromes, and the limited ED variables investigated in Connelly et al. (2014).

To our knowledge, despite the well‐known role of early childhood adversity in increasing the risk for ED, no previous studies have investigated gene–environment interactions between *OXT‐R* SNPs and poor parenting. We therefore aimed to determine: (i) whether polymorphisms in the *OXT‐R* system genes were associated with ED behaviours amongst adult women from a community sample, and (ii) whether these polymorphisms showed an interaction with poor maternal care in childhood. We hypothesized that *OXT‐R* gene polymorphisms, previously shown to be associated with ED and ED behaviours, would be associated with ED behaviours, in particular binge/purge type behaviours in this community sample of women. Given the role of OXT in affiliation and bonding, we expected an interaction between *OXT‐R* genotype and poor maternal care in increasing the risk for ED behaviours.

Because of the known overlap and crossover of syndromes and behaviours in ED, and the potential anorexigenic effect of OXT, we were particularly interested in: (i) determining the associations between *OXT‐R* SNPs and specific ED behaviours (i.e. food approach and compensatory behaviours vs. restrictive type behaviours); and (ii) if any associations became evident, taking into account the overlap between ED behaviours under study.

## Materials and methods

### Sample

ALSPAC is a longitudinal, population‐based, extensive prospective study of women and their children, set up in the 1990s to investigate the effects of environment, genetics, and other factors on child health and development (Boyd et al., 2013; Golding, Pembrey, Jones, & ALSPAC Team, 2001). All pregnant women living in the geographical area of Avon, UK, who were expected to deliver their baby between 1 April 1991 and 31 December 1992 were invited to take part in the study. ALSPAC recruited 14 541 pregnant women resident in Avon, UK with expected dates of delivery 1 April 1991 to 31 December 1992. Of these *initial* pregnancies, there was a total of 14 676 fetuses, resulting in 14 062 live births and 13 988 children who were alive at 1 year of age. Women and their children have been followed up since. Please note that the study website contains details of all the data that is available through a fully searchable data dictionary: <http://www.bris.ac.uk/alspac/researchers/data‐access/data‐dictionary/>

Women who were still enrolled in the ALSPAC study in 2009/2010 took part in a two‐phase sub‐study to investigate the prevalence of lifetime ED and ED behaviours (for details see Micali et al., submitted). Amongst these, 5658 participated. Detailed procedure and methods are described in Micali et al. (submitted) and Kothari, Barona, Treasure, and Micali (2015).

## ED behaviours

Data on ED behaviours were obtained using the ED section of the Structured Clinical Interview for DSM‐IV‐TR disorders (SCID‐I) (First, Spitzer, Gibbon, & Williams, 2002), a semi‐structured interview to diagnose Axis I DSM‐IV‐TR disorders (American Psychiatric Association, 2000). The SCID was supplemented with a version of the LIFE interview (Keller et al., 1987) adapted to ED (Anderluh, Tchanturia, Rabe‐Hesketh, Collier, & Treasure, 2009) aimed at investigating presence, frequency and duration of ED behaviours (restrictive eating, fasting, excessive exercise, binge eating, and purging), and BMI over the life course (for details see Micali et al., submitted). Women were asked to anchor their responses using major events, such as the birth of their child or other major life events in order to increase the accuracy of reporting and minimize reporting bias. Each ED behaviour was recorded over the lifetime from its first occurrence to time of the interview.

Lifetime ED behaviours: purging, restrictive eating, and binge eating obtained from interviews were coded as binary variables. Restrictive eating: women who had, at any time in their life, engaged in dietary restriction or fasting to lose weight or to avoid gaining weight (at a frequency of at least one whole day a week of severe restriction or missing a minimum of three meals a week (fasting) for a period of at least 3 months), and had not engaged at any point in their life in binge eating. Purging: women who had, at any time in their life, engaged in purging behaviours (i.e. vomiting/abuse of laxatives, diuretics, or slimming pills) at a frequency of at least once a week for a period of at least 3 months, but had never engaged in restrictive eating. Binge‐eating: women who had, at any time in their life, engaged in binge eating behaviours (overeating with loss of control) at a frequency of at least once a week for a period of at least 3 months, but had never engaged in restrictive eating.

Three hundred and ninety‐four (*n* = 394) women reported any of the ED behaviours above.

Given the high sensitivity (97.3%) of the Phase 1 screening (Micali et al., submitted), all women who screened negative in Phase 1 acted as controls (*n* = 4708). Women who participated to the ED sub‐study were overall more likely to have received secondary education and less likely to have had prior pregnancies (Micali et al., submitted).

## Maternal care

The *Maternal care score* was obtained from the maternal care subscale of the Parental Bonding Instrument (PBI) (Parker, Tupling, & Brown, 1979) completed by women at 18 weeks gestation, following enrollment in the ALSPAC cohort. This instrument assesses the quality of relationships and bond with both mother and father, up to 16 years of age, and has been shown to be reliable and valid (Wilhelm, Niven, Parker, & Hadzi‐Pavlovic, 2005). The subscale included items that measured the woman's perception of the relationship she had with her own mother—the higher the score the warmer the relationship. We categorized this variable as a binary variable, following sensitivity analyses (Micali et al. submitted) qualifying the bottom quartile (women with lowest scores), as those with poor maternal care (received from their mother).

## Genotyping

ALSPAC mothers were genotyped using the Illumina Human660W‐quad array at Centre National de Génotypage (CNG), and genotypes were called with Illumina GenomeStudio. PLINK (v1.07) was used to carry out quality control measures on an initial set of 10 015 subjects and 557 124 directly genotyped SNPs. SNPs were removed if they displayed more than 5% missingness or a Hardy–Weinberg equilibrium *p* value of less than 1.0e − 06. Additionally, SNPs with a minor allele frequency of less than 1% were removed (Fatemifar et al., 2013). Samples were excluded if they displayed more than 5% missingness, had indeterminate X chromosome heterozygosity, or extreme autosomal heterozygosity. Samples showing evidence of population stratification were identified by multidimensional scaling of genome‐wide identity by state pairwise distances using the four HapMap populations as a reference, and then excluded. Related subjects that passed quality control thresholds were retained during subsequent phasing and imputation. Individuals were imputed to HapMap Phase II (Build 36, release 22) using the Markov Chain Haplotyping software (MACH v.1.0.16)(Y. Li, Willer, Ding, Scheet, & Abecasis, 2010). Altogether, 9048 subjects and 526 688 SNPs passed these quality control filters.

We studied two genetic polymorphisms in the *OXT‐R* gene, rs2254298 and rs53576; both have three levels that represent genotypes GG, AG, and AA. The *OXT‐R* gene is located on chromosome 3 p25. It spans 19 206 base‐pairs and contains four exons and three introns. Within this gene rs53576 and rs2254298 are the two genetic variants (*D*′ = 0.64, *r*2 = 0.024), situated in the third intron, that have often been investigated. Although these variants have not been found to have any clear functional impact on the gene they may be in linkage disequilibrium with a yet unidentified functional SNP (Connelly et al., 2014). As in previous studies, we categorized the two genotypes into a dichotomous variable (GG vs. A allele carrier, with AG and AA combined) because of the small number of AA individuals. Genotype distribution (and frequency) obtained for rs53576 was as follows: G/G (*n* = 267, 48.8%), A/G (*n* = 226, 41.3%), and A/A(*n* = 54, 9.9%) (MAF = 0.41). This distribution does not deviate from the Hardy–Weinberg equilibrium, χ^2^(1) = 0.369, *p* = 0.54. Genotype distribution (and frequency) obtained for rs2254298 was: G/G (*n* = 421, 77.0%), A/G (*n* = 116, 21.2%), and A/A(*n* = 10, 1.8%) (MAF = 0.214). This distribution does not deviate from the Hardy–Weinberg equilibrium, χ^2^(1) = 0.369, *p* = 0.54. The genotype of rs2254298 was imputed using results from the GWAS analysis. Validity of the imputation was shown in Connelly et al. (2014).

In total, genotype results were available for 8330 and 8340 women for rs53576 and rs2254298.

## Covariates

Body mass index (BMI) [weight (kg)/height(m)^2^] was obtained from measured weight and height during a face to face assessment carried out contemporaneously to the two‐phase study. BMI was available on 3504 women.

Ethical approval for the study was obtained from the ALSPAC Ethics and Law Committee and the Local Research Ethics Committees.

### Statistical analyses

SNP and haplotype association analyses were performed with a logistic regression model using the SNPstats programme (Sole, Guino, Valls, Iniesta, & Moreno, 2006). All inheritance models were evaluated, and the dominant model was found to be the best fit in terms of the AIC for both rs53576 and rs2254298 (Leng et al., 2008; Bakermans‐Kranenburg & van Ijzendoorn, 2008).

We also tested the effect of the *OXT‐R* genotypes in adjusted logistic regression models where lifetime ED behaviours (binge eating, purging, restrictive eating) were the outcomes, and BMI was included as a covariate. Post‐hoc analyses investigated the effect of binge eating, purging, and their overlap (binge eating and purging). Interaction analyses between *OXT‐R* genotypes and low maternal care were carried out including an interaction term (GxE) as a predictor. Univariable, multivariable, and interaction analyses were carried out in STATA 13.

## Results

In total, data on ED behaviours was available on 5102 women, and 3698 women had complete data on ED behaviours and had valid genotype data. Missing data on BMI and maternal care restricted the sample for multivariable analyses to 3135 women. Women who had complete data on ED behaviours and genotype were slightly older at enrollment (mean age = 29.6 (SD = 4.4)), were more likely to have completed secondary education or higher (50.4%) compared to those women who had missing data on genotype (respectively, mean age = 29.2 (SD = 4.7), completed A‐levels or degree (40.3%)).

The *OXT‐R* rs53576 A allele was negatively associated with binge eating and purging (respectively: odds ratio (OR) = 0.52 (95%confidence intervals (CI): 0.36–0.76), OR = 0.66 (95%CI: 0.47–0.92)), suggesting a protective effect. In contrast, the rs2254298 A allele was positively associated with purging behaviours (OR = 1.50 (95%CI: 1.03–2.19), *p* = 0.03) and restrictive eating (OR = 2.14 (95%CI: 1.42–3.23), *p* = 5.1 × 10^−4^).

Haplotype association analyses showed that the rs53576 rs2254298 GA haplotype was positively associated with increased odds of restrictive eating (OR = 2.04 (1.41–2.96), *p* = 2 × 10^−4^) and purging (OR = 1.92 (1.06–3.47), *p* = 0.03) (see Table 1).

**Table 1:** Associations between *OXT‐R* haplotypes and ED behaviours amongst 3535 women

*OXTR haplotypes*		Haplotype frequency	Restrictive eating		Binge eating		Purging	
			OR (95%CI)	*p* Value^a^	OR (95%CI)	*p* Value^a^	OR (95%CI)	*p* Value^a^
rs53576	rs2254298							
G	G	(*n* = 2019) 57%	Ref.	**0**.**99 × 10** ^−**3**^	Ref	**0**.**006**	Ref.	**0**.**01**
A	G	(*n* = 1119) 31.7%	0.89 (0.64–1.23) *p* = 0.47		**0**.**69** (**0**.**51–0**.**94**) ***p*** = **0**.**02**		0.84 (0.64–1.11) *p* = 0.21	
G	A	(*n* = 348) 9.9%	**2**.**04** (**1**.**41–2**.**96**) ***p*** = **2 × 10** ^−**4**^		1.17 (0.79–1.74) *p* = 0.42		**1**.**47** (**1**.**03–2**.**08**) ***p*** = **0**.**03**	
A	A	(*n* = 47) 1.3%	N/A		N/A		N/A	

a *p* Value for trend.

Bold indicates significant associations.

In BMI‐adjusted multivariable analyses, the rs53576 GG genotype carriers had increased odds of binge eating (OR = 1.91 (1.31–2.78), *p* = 0.001) and purging (OR = 1.55 (1.09–2.22), *p* = 0.01). Restrictive eating was not associated with rs53576 genotype (Table 3). The rs2254298 AG/AA genotype carriers had increased odds of restrictive eating (OR = 2.23 (1.42–3.49), *p* < 0.0001), but not of binge eating or purging.

Poor maternal care was independently associated with both binge eating and purging (respectively, OR = 1.65 (1.10–2.46), *p* = 0.01; OR = 1.67 (1.02–2.72), *p* = 0.04).

When the interaction term was added to the model, there was no significant interaction between *OXT‐R* genotype for either rs53576 and rs2254298 and poor maternal care in respect to all three outcomes (Table 2). There was a reduction in magnitude of the effect of rs53576 GG genotype (vs. AA/AG genotype) in relation to binge eating when including the interaction term (Table 2). Similarly, the magnitude of the effect of rs2254298 genotype in relation to restrictive eating decreased when including the interaction term.

**Table 2:** Logistic regression models of associations between ED behaviours and rs53576 genotype and low maternal care (model 1), and their interaction (model 2): odds ratios (and 95% confidence intervals) adjusted by BMI^a^ amongst 3171 women

	*Restrictive eating* *OR* (*95*%*CI*)	*Binge eating* *OR* (*95*%*CI*)	*Purging* *OR* (*95*%*CI*)
*Model 1*			
rs53576 GG genotype^b^	1.50 (0.99–2.28)	1.91***(1.31–2.78)	1.55** (1.09–2.22)
Low maternal care^c^	1.64 (0.85–3.14)	1.40* (1.00–1.97)	1.41* (1.01–1.96)
*Model 2*			
rs53576 GG genotype^b^	1.43 (0.87–2.34)	1.52* (1.00–2.33)	1.62* (1.01–2.43)
Low maternal care^c^	1.24 (0.58–2.63)	1.38 (0.73–2.62)	1.76* (1.00–3.14)
rs53576 GG genotype × poor maternal care	1.16 (0.42–3.17)	1.34 (0.59–3.06)	0.86 (0.39–1.87)
*Model 1*			
rs2254298 AG/AA genotype^d^	2.23*** (1.42–3.49)	1.24 (0.81–1.93)	1.47 (0.97–2.23)
Poor maternal care^c^	1.41 (0.86–2.33)	1.76**(1.17–2.68)	1.53* (1.00–2.34)
*Model 2*			
rs2254298 AG/AA genotype^d^	2.08**(1.23–3.49)	1.13 (0.67–1.91)	1.19 (0.72–1.96)
Poor maternal care^c^	1.28 (0.69–2.38)	1.62* (1.00–2.63)	1.22 (0.73–2.04)
rs2254298 AG/AA genotype × poor maternal care	1.33 (0.47–3.79)	1.41 (0.54–3.65)	2.23 (0.89–5.62)

* *p* ≤ 0.05.

** *p* ≤ 0.01.

*** *p* ≤ 0.001.

a *n* = 3022 women with no lifetime ED behaviours are the referent group.

b AG/AA genotype is the referent group.

c Lowest quartile vs. remaining 75%.

d GG genotype is the referent group.

Given the observed association between *OXT‐R* genotypes and binge/purge behaviours, post‐hoc analyses explored the association between the *OXT‐R* rs53576 and rs2254298 genotypes, poor maternal care and their interaction and the occurrence of binge eating in the absence of purging, purging in the absence of binge eating, and their overlap (binge eating and purging lifetime). As shown in Table 3, rs53576 GG genotype carriers had two‐fold increased odds of having experienced both binge eating and purging lifetime (OR = 1.94 (1.13–3.34)) compared to AA/AG carriers, and this association increased in magnitude when the interaction term was included in the model (OR = 3.02 (1.43–6.38)); however, the interaction between genotype and poor maternal care was not significant. In contrast, although there was a trend in rs2254298 A‐allele carriers having higher odds of both binge eating and purging, the interaction between this genotype and poor maternal care was significant (*p* = 0.03), and women with both the rs2254298 AG/AA genotype and poor maternal care had a four‐fold increased odds of binge eating and purging (OR = 4.40 (1.11–17.4)) (see Table 3).

**Table 3:** Post‐hoc associations between binge eating, purging and their co‐occurrence, OXTR genotypes, low maternal care, and their interaction adjusted by BMI (*n* = 3008)^a^

*Binge eating and purging* (*n* = *83*, *1*.*65*%)		*Binge eating* (*n* = *103*, *2*.*05*%)	*Purging* (*n* = *124*, *2*.*46*%)	*Controls* (*n* = *2823* b)
rs53576 GG genotype^c^	**3**.**02** (**1**.**43–6**.**38**) ***p*** = **0**.**004**	1.37 (0.77–2.45) *p* = 0.28	1.65 (0.92–2.95) *p* = 0.09	Ref
Low maternal care^d^	1.84 (0.57–5.92) *p* = 0.30	1.58 (0.69–3.57) *p* = 0.27	**2**.**23** (**1**.**06–4**.**70**) ***p*** = **0**.**03**	Ref
rs53576 GG genotype × low maternal care	0.87 (0.21–3.54) *p* = 0.84	1.28 (0.43–3.77) *p* = 0.65	0.31 (0.09–1.04) *p* = 0.06	Ref
rs2254298 AG/AA genotype^e^	1.18 (0.53–2.64) *p* = 0.68	1.13 (0.57–2.23) *p* = 0.72	1.28 (0.66–2.48) *p* = 0.47	Ref
Low maternal care^d^	1.01 (0.41–2.48) *p* = 0.97	2.09 (1.17–3.73) *p* = 0.01	1.28 (0.66–2.49) *p* = 0.46	Ref
rs2254298 AG/AA genotype × low maternal care	**4**.**40** (**1**.**11–17**.**4**) ***p*** = **0**.**03**	0.42 (0.08–2.14) *p* = 0.29	1.22 (0.32–4.68) *p* = 0.77	Ref

a Behaviours are mutually exclusive; women with restrictive eating were excluded.

b Three women had missing data on rs2254298 genotype.

c AG/AA genotype is the referent group.

d Lowest quartile vs. remaining sample.

e GG genotype is the referent group.

Bold indicates significant associations.

## Discussion

This study focused on investigating whether candidate genetic variants in the oxytocin receptor system (*OXT‐R*), well‐studied in the context of social behaviour, were associated with ED behaviours and interacted with poor maternal care in increasing the risk for ED behaviours in a large population‐based study of women (ALSPAC). We focused on the two most studied SNPs in the oxytocin receptor gene (rs2254298 and rs53576). Both rs2254298 and rs53576 showed associations with ED behaviours. Women who were homozygotes for the G allele in the rs53576 SNP were more likely to have had lifetime binge eating and purging behaviours; once we accounted for the co‐occurrence of these behaviours, this association became stronger. These findings are in line with findings from Kim et al (Kim, Kim et al., 2015), who showed an association between the same genotype and BN in a clinical sample. Our findings also extend recent findings from ALSPAC (Connelly et al., 2014) that highlighted an association between rs53576 GG homozygosity and self‐reported purging using a hypothesis‐free approach.

Association patterns between ED behaviours and rs2254298 differed compared to rs53576. The rs2254298 A allele carriers were more likely to have experienced restrictive eating over their lifetime. Although women with AA genotype did not have higher odds of binge eating or purging, women with AG/AA genotypes in the presence of poor maternal care had a four‐fold increase in the odds of binge eating and purging. The role of the two SNPs under study was further corroborated by haplotype analyses showing that women with the OXTR rs53576 rs2254298 GA haplotype had increased odds of restrictive eating and purging.

There is increasing evidence that oxytocin is involved not only in the regulation of food intake, but also in ED. Oxytocin secretion has been shown to be dysregulated in low‐weight and weight‐recovered individuals with AN (Afinogenova et al., in press; Lawson et al., 2011; Lawson et al., 2012). Moreover, abnormal postprandial oxytocin levels in AN before and after weight recovery are associated with reduced fMRI activation of food motivation neural circuitry and increased severity of ED psychopathology (Lawson et al., 2012). In addition to the evidence from animal studies that oxytocin administration affects appetitive behaviour, oxytocin administration to healthy men reduced caloric consumption, particularly of palatable foods (e.g. fats, carbohydrates) without changing subjective appetite, implicating reward pathways as potential candidate mechanisms (Kim, Eom, Yang, Kang, & Treasure, 2015; Lawson et al., 2015; Ott et al., 2013).

The available literature on *OXT‐R* genetic variants and behaviour, especially in relation to psychiatric outcomes, is mixed. Some authors have hypothesized that mixed findings are a reflection of polymorphic variation in *OXT‐R* genes not being a risk marker per se, but markers of differential susceptibility once environmental risk factors are included in the mix (Brune, 2012). Therefore, although GG rs53576 homozygotes are usually considered to have higher empathy (Rodrigues et al., 2009), higher optimism and self‐esteem (Saphire‐Bernstein et al., 2011), and respond more sensitively to their children (Bakermans‐Kranenburg & van Ijzendoorn, 2008), they might also be ‘differentially susceptible’ to adversity, that is, have better outcomes in a nurturing environment but worse outcomes in the presence of adversity. In fact, G allele carriers have been shown to be more likely to develop depression and emotional dys‐regulation in the context of early adversity (Bradley et al., 2011; McQuaid et al., 2013). Similarly, previous studies have shown an interaction between rs2254298 polymorphisms (A carriers) and early adversity in respect to depressive symptoms, greater social anxiety, and physical symptoms of anxiety (Thompson, Parker, Hallmayer, Waugh, & Gotlib, 2011). Our study showed a similar effect modification in relation to bulimic symptoms.

Finally, although both SNPs under study are in intronic regions of the *OXT‐R* gene, not leading to functional changes in the *OXT‐R*, there is evidence that rs2254298 polymorphisms are associated with biological differences. For example, lower plasma levels of OXT have been identified amongst GG carriers (Feldman et al., 2012). Volume of the amygdala has been shown to increase with increasing number of A alleles of rs2254298 (Furman, Chen, & Gotlib, 2011; Inoue et al., 1994). Functional and structural brain differences of rs53576 polymorphisms were also highlighted amongst healthy subjects, with GG homozygotes having higher activation of the amygdala during a face emotion processing task, and lower coupling of hypothalamus/amygdala connectivity (Tost et al., 2010). It is possible that these intronic *OXT‐R* gene polymorphisms might therefore have a regulatory function, cause altered splicing (Cooper, 2010), or influence transcriptional activity or methylation of nearby genomic regions (Cooper, 2010); or that they might be a marker of a yet unidentified functional polymorphism.

These findings need to be taken in context of relevant strengths and limitations. There are several strengths to this study. First, it relied on a large sample; second, the population‐based nature of the sample allowed us to focus on behaviours, likely to yield more precise patterns of biological markers compared to diagnostic syndromes (Anderluh et al., 2009). Data on ED behaviours were collected using validated, well‐tested instruments in mid life, allowing us to capture presence of distinct ED behaviours and their co‐occurrence across most of the women's life span. Data on maternal care were obtained 20 years prior to the ascertainment of ED behaviours, therefore decreasing the likelihood of reporting bias. All analyses were adjusted by objectively measured BMI, therefore ensuring that associations were not confounded by BMI. Limitations include the relatively low prevalence of some ED behaviours, leading to low power to detect small associations and therefore possible false negatives. To be included in ALSPAC, women had to be able to become pregnant; it is therefore possible that this sample is representative of women with less severe ED. However, as highlighted in (Micali et al., submitted), a range of ED severity was evident across the sample. Similarly, our findings might not generalize to all women; Connelly et al. (2014), however, found limited evidence of associations between the SNPs under study and reproductive characteristics, suggesting that these are unlikely to confound any association seen. We chose to focus on maternal care as a more global measure of parenting rather than more extreme forms of maltreatment, which have low prevalence and are more difficult to ascertain; therefore, our findings cannot be extrapolated to more severe maltreatment.

This study replicates and extends previous findings of an association between OXTR gene polymorphisms and ED behaviours. It is the first study to demonstrate an interaction between poor maternal care and an *OXT‐R* polymorphism (rs2254298) in increasing the risk for bulimic type behaviours. Given our increased understanding of the role of the oxytonergic system in appetite and eating behaviour, these findings lay an important foundation to further its study in the context of ED and eating behaviours.
